# Proteomic Responses Under Cold Stress Reveal Unique Cold Tolerance Mechanisms in the Pacific White Shrimp (*Litopenaeus vannamei*)

**DOI:** 10.3389/fphys.2018.01399

**Published:** 2018-11-13

**Authors:** Jin-Xia Peng, Ping-Ping He, Pin-Yuan Wei, Bin Zhang, Yong-Zhen Zhao, Qiang-Yong Li, Xiu-Li Chen, Min Peng, Di-Gang Zeng, Chun-Ling Yang, Xiaohan Chen

**Affiliations:** Guangxi Key Laboratory of Aquatic Genetic Breeding and Healthy Aquaculture, Guangxi Academy of Fishery Sciences, Nanning, China

**Keywords:** *Litopenaeus vannamei*, cold stress, differentially expressed proteins, qRT-PCR, proteomics

## Abstract

The Pacific white shrimp (*Litopenaeus vannamei*), one of the most widely cultured shrimp species in the world, often suffers from cold stress. To understand the molecular mechanism of cold tolerance in Pacific white shrimp, we conducted a proteomic analysis on two contrasting shrimp cultivars, namely, cold-tolerant Guihai2 (GH2) and cold-sensitive Guihai1 (GH1), under normal temperature (28°C), under cold stress (16°C), and during recovery to 28°C. In total, 3,349 proteins were identified, among which 2,736 proteins were quantified. Based on gene ontology annotations, differentially expressed proteins largely belonged to biological processes, cellular components, and molecular functions. KEGG pathway annotations indicated that the main changes were observed in the lysosome, ribosomes, and oxidative phosphorylation. Subcellular localization analysis showed a significant increase in proteins present in cytosol, extracellular regions, and mitochondria. Combining enrichment-based clustering analysis and qRT-PCR analysis, we found that glutathione S-transferase, zinc proteinase, m7GpppX diphosphatase, AP2 transcription complex, and zinc-finger transcription factors played a major role in the cold stress response in Pacific white shrimp. Moreover, structure proteins, including different types of lectin and DAPPUDRAFT, were indispensable for cold stress tolerance of the Pacific white shrimp. Results indicate the molecular mechanisms of the Pacific white shrimp in response to cold stress and provide new insight into breeding new cultivars with increased cold tolerance.

## Introduction

The Pacific white shrimp (*Litopenaeus vannamei*) is a widely cultured species in subtropical areas such as China as well as many other Asian countries. Many biotic factors affect the growth and yield of Pacific white shrimp, such as early mortality syndrome (EMS) (Chuchird et al., 2015), Taura syndrome virus (TSV) (Zeng et al., 2013), associated bacterial communities (Zheng et al., 2017), and *Spiroplasma penaei* sp. nov., a bacterial species associated with mortality (Nunan et al., 2005). Abiotic stresses also considerably influence the aquaculture of these organisms. These stresses include low temperatures, which can cause persistent effects on fish muscle (Schnurr et al., 2014), and acute ammonia stress, which can lead to the death of the Pacific white shrimp (Heisterkamp et al., 2016; Lu et al., 2016).

In recent years, farming areas in China have been significantly expanded to meet the increasing demands for the Pacific white shrimp. However, cultivation in the subtropical areas of China, including the Guangdong Province, the Guangxi Zhuang Autonomous Region, the Hainan Province, and Northern China, is adversely and frequently affected by cold stress (i.e., water temperature below 16°C). Low water temperature causes growth retardation, digestion malfunction, and energy metabolism disorders in the Pacific white shrimp. Breeding of new shrimp cultivars with cold tolerance is, therefore, urgently required (Peng et al., 2015). However, the conventional generation cycle of the Pacific white shrimp is long and the breeding efficiency is extremely low (Castillo-Juarez et al., 2015). Although cold resistance in organisms is usually controlled by quantitative traits associated with multiple physiological and biochemical processes, identifying major genes conferring cold resistance is very important. Zinc finger-containing glycine-rich RNA-binding proteins confer cold tolerance in rice (Kim and Kang, 2006; Chaikam and Karlson, 2008; Park et al., 2009). Recently, transcriptomic, proteomic, and metabolomic studies have been performed to effectively investigate stress tolerance in animals and plants (Chen et al., 2008; Kowalczuk et al., 2018). For example, in the Pacific white shrimp, high-throughput sequencing was used to identify the miRNAs that respond to white spot syndrome virus (WSSV) infections (Zeng et al., 2015) and in organisms under acute ammonia stress (Lu et al., 2016). To reveal the molecular mechanisms of cold tolerance in Pacific white shrimp, a cold-tolerant cultivar GH2 and a cold-sensitive cultivar GH1 were investigated under normal temperature, low temperature, and in recovery stages using proteomics, bioinformatics, and qRT-PCR techniques. The results provide new insight into the molecular mechanisms of the Pacific white shrimp associated with cold stress to facilitate the breeding of new cultivars with increased cold tolerance.

## Materials and Methods

### Pacific White Shrimp Cultivars and Treatment

Two Pacific white shrimp cultivars, Guihai2 (GH2) with cold tolerance and Guihai1 (GH1) with high yields but sensitive to cold stress, were reared at Fangchenggang Aquaculture Base of the Guangxi Academy of Fishery Sciences (E108°41′62″, N21°61′30″). GH1 is a high-yield cultivar developed by the Guangxi Academy of Fishery Sciences in 2013. Since 2015, the acclimation conditions for cold-tolerant cultivar breeding have included exposure to 16°C for 144 h, and these breeding conditions for the cold-resistant cultivar GH2 result in its resistance to low temperatures. GH2 shrimp grew well at 20–22°C and the low-temperature resistance improved by 3–5°C, and its adaptability to variable temperatures enhanced significantly. Organisms were adapted to conditions of 28°C, 32–35% salinity, and a dissolved oxygen level of ≥5 mg L^−1^ for 1 week prior to experimentation. Organisms used were 3 months old with a weight of 10–15 g. Shrimp were fed three times daily, with a daily ration of approximately 5% of the total body weight of a shrimp. Two groups of shrimp were cooled to specific experimental temperatures and then maintained at those temperatures for sampling at different points. The control group was maintained at 28°C. To acclimate the experimental groups to a lower temperature, the temperature was lowered to 16°C, which was maintained for 144 h, with feeding halted only in the experimental groups from this point forward. After 144 h, one group was allowed to recover to 28°C (Table 1). Three organisms from each group were sampled, with shrimp sacrificed by destroying the main center nerve and rapidly isolating the hepatopancreas for subsequent protein extraction (He et al., 2018). Each of the three group treatments were repeated twice. Reproducibility analysis of the two repeated trials was conducted by Pearson correlation coefficient methods.

**Table 1:** Samples name of proteome analysis.

Cultivar	Temperature	Proteome group abbreviation	Temperature groups abbreviation
GH2	28°C	GH2-28	GH2GH1-28
GH1	28°C	GH1-28	
GH2	16°C	GH2-16	GH2GH1-16
GH1	16°C	GH1-16	
GH2	Recovery from 16 to 28°C	GH2-R	GH2GH1-R
GH1	Recovery from 16 to 28°C	GH1-R	

**FIGURE 1:**
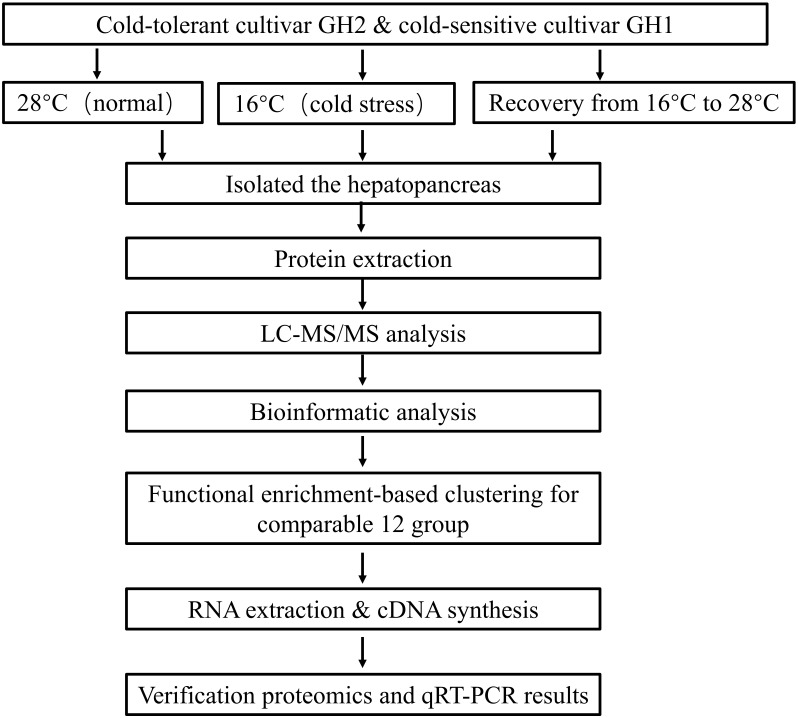
Schematic diagram of technical route of treatment, proteomic analysis and verification.

All the identified proteins were compared among the two cultivars under the different temperature treatments. The experimental procedure is shown in Figure 1. The two shrimp cultivars were treated at three temperature levels (Table 1). Differentially expressed proteins were cross-compared in 12 groups, namely: 1. GH2-28 vs. GH1-28, 2. GH2-28 vs. GH2-16, 3. GH2-R vs. GH2-28, 4. GH2-16 vs. GH1-16, 5. GH2-16 vs. GH2-R, 6. GH2-R vs. GH1-R, 7. GH1-16 vs. GH1-28, 8. GH1-R vs. GH1-28, 9. GH1-16 vs. GH1-R, 10. GH2GH1-16 vs. GH2GH1-28 (hereafter abbreviated as 16 vs. 28), 11. GH2GH1-R vs. GH2GH1-28 (hereafter abbreviated as R vs. 28), and 12. GH2GH1-R vs. GH2GH1-16 (hereafter abbreviated as R vs. 16).

### Proteomic Analysis

Shrimp samples were sonicated three times on ice using a high-intensity ultrasonic processor (Ningbo Scientz Biotechnology Co., Ltd., Ningbo, China) in lysis buffer [8 M urea, 2 mM (Ethylenedinitrilo)tetraacetic acid (EDTA), 10 mM DL-Dithiothreitol (DTT), and 1% Protease inhibitor cocktail]. The remaining debris was removed by centrifugation at 20,000 *g* at 4°C for 10 min. Finally, protein was precipitated with cold 15% TCA for 2 h at −20°C. After centrifugation at 4°C for 10 min, the supernatant was discarded, and the remaining precipitate was washed three times with cold acetone. Protein precipitate was redissolved in buffer [8 M urea, 100 mM Triethylammonium bicarbonate (TEAB), pH 8.0], and the protein concentration was determined by using a 2-D Quant Kit (GE Healthcare, Beijing, China) according to the manufacturer’s instructions. For trypsin digestion, protein samples were diluted by adding 100 mM TEAB to a urea concentration less than 2 M. Finally, trypsin was added at a 1:50 trypsin-to-protein mass ratio for the first digestion overnight and a 1:100 trypsin-to-protein mass ratio for a second 4 h-digestion. After trypsin digestion, peptides were desalted by using a Strata X C18 SPE column (Phenomenex, Tianjin, China) and vacuum-dried for tandem mass tag (TMT) labeling. Samples were mixed and then fractionated by using high-pH reverse-phase high-performance liquid chromatography (HPLC) using an Agilent 300 Extend C18 column (5 μm particles, 4.6 mm ID, 250 mm length). Peptides were dissolved in 0.1% FA and directly loaded onto a reversed-phase precolumn (Acclaim PepMap 100, Thermo Fisher Scientific, Shanghai, China). Peptide separation was performed using a reversed-phase analytical column (Acclaim PepMap RSLC, Thermo Fisher Scientific, Shanghai, China). The gradient comprised an increase from 6 to 23% solvent B (0.1% FA in 98% ACN) over 26 min, 23 to 35% in 8 min and climbing to 80% in 3 min, and then holding at 80% for the last 3 min, all at a constant flow rate of 400 nL min^−1^ on an EASY-nLC 1000 ultra performance liquid chromatography (UPLC) system. The peptides were subjected to an NSI source followed by tandem mass spectrometry (MS/MS) in Q Exactive^TM^ plus (Thermo Fisher Scientific, Shanghai, China) coupled online to the UPLC.

### Bioinformatic Analysis

#### Protein Annotation and Subcellular Localization

Gene ontology (GO) annotations were created by searching the UniProt-GOA database^1^. The functional description of protein domains were annotated by using the InterProScan tool^2^ based on protein sequence alignment; the InterPro domain database was also used. Subcellular localization was performed using the WoLF PSORT online software^3^.

#### GO Enrichment Analysis

Proteins were classified by GO annotation into three categories: biological process, cellular compartment, and molecular function. For each category, a two-tailed Fisher’s exact test was employed to test the enrichment of the differentially expressed protein against all the identified proteins. Correction for multiple hypothesis testing was carried out using standard false discovery rate (FDR) control methods. A GO with a corrected *p-*value < 0.05 was considered to be significant.

#### Pathway Enrichment Analysis

The Kyoto Encyclopedia of Genes and Genomes (KEGG) database^4^ was used to identify the enriched pathways by a two-tailed Fisher’s exact test to investigate the enrichment of the differentially expressed protein against all identified proteins. Correction for multiple hypothesis testing was carried out using standard false discovery rate control methods. The pathway with a corrected *p-*value < 0.05 was considered significant. These pathways were classified into hierarchical categories according to the KEGG website.

#### Protein Domain Enrichment Analysis

For each category of protein, InterPro, a database resource that provides a functional analysis of protein sequences by classifying them into families and predicting the presence of domains and important sites^5^ was searched and a two-tailed Fisher’s exact test was employed to test the enrichment of the differentially expressed protein against all identified proteins. Correction for multiple hypothesis testing was carried out using standard false discovery rate control methods and domains with a corrected *p-*value < 0.05 were considered significant.

#### Comparative Clustering Enrichment Analysis

All the protein groups obtained after enrichment were collated, along with their *p*-values, and then filtered for categories that were at least enriched in one of the clusters with *p-*value < 0.05. This filtered *p*-value matrix was transformed by the function x = −log10 (*p*-value). Finally, these x values were z-transformed for each category. The z scores were then clustered by using one-way hierarchical clustering (Euclidean distance, average linkage clustering) in Genesis Software. Cluster membership was visualized by a heat map using the “heatmap.2” function from the “gplots” R-package. All the data related to this study are available on iProX^6^ with id IPX0001223000/PXD009889.

### RNA Extraction and cDNA Synthesis

Total RNA was extracted from the two shrimp cultivars (18 samples) using the RP5611 RNA Rapid Extraction Kit (BioTeke Corporation, Beijing, China). The quality and concentration of the extracted RNA were determined by agarose gel electrophoresis and measured by using a spectrophotometer. First-strand cDNA was synthesized from 2 mg of the total RNA with MMLV reverse transcriptase and random hexamer primer (Takara, Dalian, China) according to the manufacturer’s instructions.

### Quantitative RT-PCR (qRT-PCR) Assays

Transcriptomic analysis of the same samples (data not published) was performed in addition to protein identification. The ID numbers of proteins were the same as that of the transcriptome sequence. After selecting the protein to be verified according to the results of the differential analysis, the transcriptome sequence with the corresponding ID number was retrieved for primer design and verification. Based on proteomic analysis, 18 differentially expressed proteins under cold stress were selected for designing primers and qRT-PCR verifications in the two shrimp cultivars. The primers of the selected genes were designed by using Primer Premier 6 (PREMIER Biosoft, Palo Alto, CA, United States) and synthesized by GENEWIZ Biotechnology (Suzhou, China). The qRT-PCR assays were performed using 2× SYBR Green qPCR ProMix (Takara, Dalian, China) on an ABI 7500 Real-Time PCR System (ABI, Thermo Fisher Scientific, Shanghai, China) following the manufacturer’s instructions. Each plate was analyzed independently in triplicate for all the reference and selected genes. The beta-actin gene was used as a reference gene, and the 2^−ΔΔCt^ method (Livak and Schmittgen, 2001) was used to evaluate the relative gene expression levels. The protein accession numbers, description, and primers used in the qRT-PCR tests are listed in Table 2.

**Table 2:** Protein accession, description and qRT-PCR primer pairs.

Protein accession	Protein description	Primers (5′-3′)
CL10443Contig1	Hypothetical protein	F1:CAGCCGGTTCCGTTTTCTTG
	DAPPUDRAFT_240263	R1: CCTGCTGTGGATTTCGGTCT
SCL1Contig301	AP-2 complex subunit alpha	F1: GCACCAGCAGTACAGTTCCA
		R1: CATCAGAGGAGCGGAGGTTG
CL9159Contig1	Lectin	F1: TTCTGCCACTCGTTTCTGGG
		R1: TTGGCTCCTGGGTTTTCGAG
SCL1Contig877	Hypothetical protein	F1: GTGGTGAGGCTGTAGGTTCC
	DAPPUDRAFT_307838	R1: GCCTGGGACTTCTACGACAC
CL6694Contig3	Hypothetical protein	F1: CTGCACGTAACTCTGCTCCA
	BRAFLDRAFT_206907	R1: ATTCTGCCCCCAACATCGTC
SCL8Contig75	Tetraspanins-like protein CD63	F1: TGACATCCAAACGCCAACCA
		R1: GGCCGTAATGTGTTCTCCGT
CL23275Contig1	Zn-finger in Ran binding protein and others domain containing protein	F1: GAGATCCGAGTGCGACTGAAA
		R1: GACCTCAAGCAAGCAAGCAC
SCL12Contig31	Lectin D	F1: TCATTCAGGGGAGCCGAGAT
		R1: TTCGGGGAAGTCGCTGTTTT
CL638Contig3	C-type lectin	F1: GCCCCCATGTTAGAGCACAA
		R1: ATCTCAATCACCCAACGCCC
GH1_28_1-c16133_g2_i1	Hypothetical protein	F1: AGCTGATGGACTGCGTTCAG
	DAPPUDRAFT_321849	R1: CGAAGTCGAAGTAGGGCTGG
CL4347Contig1	Lectin	F1: CTACTCCCACCTTGGCATCG
		R1: TGAAAGTAGTGGAGGGCGGA
CL9739Contig1	Hypothetical protein	F1: AGTTCCACGGTTCCCTACCT
	DAPPUDRAFT_54086	R1: AACTTTCACCCGCACCGTAA
CL10847Contig1	Hypothetical protein	F1: GTGCCGGTTGGAGCTTCTAT
	DAPPUDRAFT_215063	R1: GATGCTCCCTGCTCGTATCC
CL3798Contig3	Zinc proteinase	F1: CCATCGGCTTCTTCCACGAG
		R1: CTTGTTGCACTCCTTCCCGT
CL5480Contig1	Hypothetical protein	F1: TGTTGCCTCATTCATCGCCT
	DAPPUDRAFT_31637, partial	R1: CAGTGGCGTTGTTGGGAATG
GH1_16_2-c14615_g2_i2	m7GpppX diphosphatase	F1: GTGTTCCTGCTGTTCGTCCT
		R1: CGATGGCTTCCTTGGGCATA
CL4762Contig4	Hypothetical protein	F1: TTGGAGCTATGCGGCTGTTT
	DAPPUDRAFT_303198	R1: CTGTGCCTGATGGAATGGGT
CL823Contig3	Lectin D	F1: GCACCAGCAGTACAGTTCCA
		R1: CATCAGAGGAGCGGAGGTTG
Van-beta-Actin	Actin	F1: GGACTTCGAGCAGGAGATGACCAC
		R1: ACGTCGCACTTCATGATGGAGTTG

## Results

**FIGURE 2:**
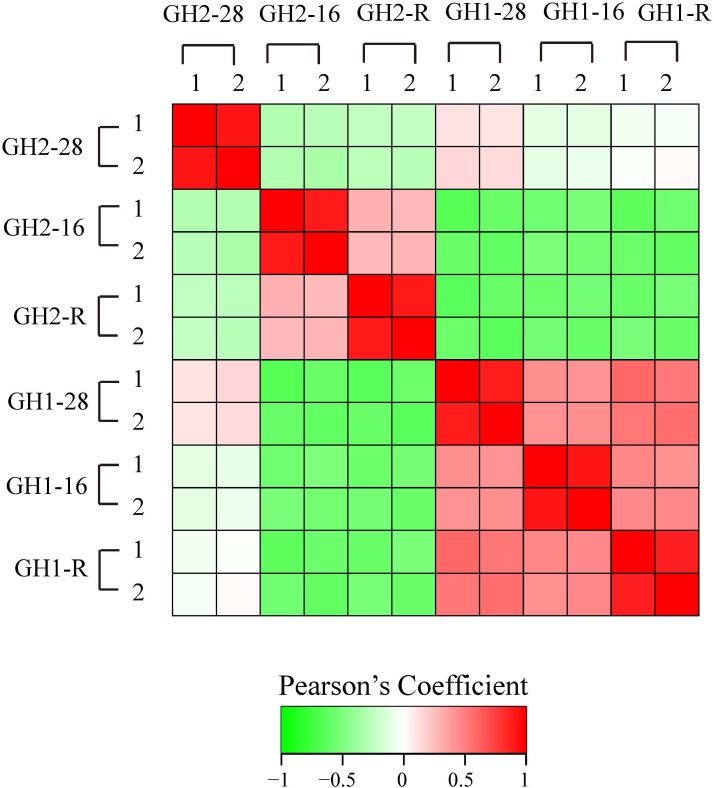
Reproducibility analysis of two repeated trials by Pearson correlation coefficient. For each replicate, relative expressions were calculated and Pearson’s correlation coefficient was calculated.

We first analyzed the proteome quality of two repeats (Figure 2). The similarity of the two repeats ranged from 0.879 in GH1-R to 0.918 in GH2-28 and GH1-16 based on Pearson correlation coefficient analysis, and these values suggested that the proteome quality was suitable for subsequent analysis.

### Proteomic Analysis Revealed Differentially Expressed Proteins Between Cold-Tolerant Cultivar GH2 and Cold-Sensitive Cultivar GH1 Under Cold Stress

In total, 3,349 identified proteins were found in the proteome, among which 2,736 proteins were quantified (Table 3 and Supplementary Tables S1, S2). The number of differential proteins between the two shrimp cultivars under the same temperature were much lower than those under different temperatures. At the normal temperature of 28°C (GH2-28 vs. GH1-28), there were 71 upregulated and 59 downregulated proteins differentially expressed between the cold-tolerant cultivar GH2 and the cold-sensitive cultivar GH1 (Supplementary Table S3). However, under the low temperature of 16°C (GH2-16 vs. GH1-16), the number of differentially expressed proteins increased to 274 upregulated and 139 downregulated proteins. With the recovery regime of 16 to 28°C (GH2-R vs. GH2-R), there were 236 upregulated and 129 downregulated proteins. These results indicated that there were different proteomic profiles in cold-tolerant and cold-sensitive cultivars, especially under the low-temperature treatments and in the recovery period.

**Table 3:** Numbers of differentially expressed proteins to be quantified between different groups.

Group No.	Group name	Up-regulated (>1.5)	Down-regulated (<1/1.5)
1	GH2-28 vs. GH1-28	71	59
2	GH2-28 vs. GH2-16	174	108
3	GH2-R vs. GH2-28	142	97
4	GH2-16 vs. GH1-16	274	139
5	GH2-16 vs. GH2-R	50	55
6	GH2-R vs. GH1-R	236	129
7	GH1-16 vs. GH1-28	50	43
8	GH1-R vs. GH1-28	28	27
9	GH1-16 vs. GH1-R	41	36
10	GH2GH1-16 vs. GH2GH1-28	35	64
11	GH2GH1-R vs. GH2GH1-28	29	40
12	GH2GH1 R vs. GH2GH1-16	19	18
	Total	1149	815

*p < 0.05.*

### The GO Distribution of Differentially Expressed Proteins

To further understand the functions and features of the identified and quantified proteins, they were classified into four categories, namely, gene ontology, domain, pathway, and subcellular localization. According to the GO annotation information of the identified proteins, the amount of the differentially expressed proteins for each GO term of level 2 was aggregated (Table 4 and Supplementary Table S4). A low temperature caused important biological process changes in the two cultivars. First, the number of proteins for metabolic processes, cellular processes, single-organism processes, localization, and biological regulation significantly increased. Second, the low temperature caused an increase in the response of proteins to stimuli. Third, the numbers of proteins for cellular component organization or biogenesis increased only in the recovery group. There were 130 proteins differentially expressed in the cold-tolerant cultivar GH2 and the cold-sensitive cultivar GH1 under normal conditions. A low temperature caused an increase in the number of proteins attributed to biological processes, especially the response of proteins to stimuli in the cold-tolerant cultivar GH2. During the recovery process, the growth of shrimp recovered, and a decrease in the number of proteins that responded to stimuli and an increase in the number of proteins attributed to cellular component organization or biogenesis was observed. In addition, 22 and 11 proteins, respectively, of an unknown function, were present in the low-temperature and recovery groups. Significant changes in the upregulated and downregulated proteins were also found according to cellular component and molecular function categories. Not only did the number of proteins associated with the cell, organelle, macromolecular complexes, and the membrane extracellular region categories increase, but the proteins associated with the extracellular regions also increased in the cold-tolerant cultivar GH2 under the low temperature.

**Table 4:** The GO distribution of all up-regulated and down-regulated proteins.

GO terms level 1	GO terms level 2	Differential expressed protein numbers								
		GH2-28 vs. GH1-28	GH2-16 vs. GH2-28	GH2-16 vs. GH1-16	GH2-R vs. GH2-28	GH2-R vs. GH2-16	GH2-R vs. GH1-R	GH1-16 vs. GH1-28	GH1-R vs. GH1-28	GH1-R vs. GH1-16
**Cellular component**	Extracellular matrix	0	0	0	0	0	0	0	0	0
	Membrane-enclosed lumen	0	1	1	1	0	1	0	0	0
	Organelle	11	27	46	24	2	37	3	1	1
	Membrane	5	29	39	12	7	28	7	1	4
	Cell junction	0	0	1	0	0	0	0	0	0
	Macromolecular complex	9	25	44	14	2	39	3	1	2
	Cell	15	37	76	36	6	64	6	1	5
	Extracellular region	3	11	11	8	1	10	6	3	5
	Synapse	0	0	0	0	0	0	0	0	0
**Molecular function**	Electron carrier activity	6	2	12	1	2	9	0	0	1
	Molecular transducer activity	0	0	2	0	0	1	0	0	0
	Catalytic activity	70	105	176	98	47	176	46	18	36
	Binding	43	123	175	99	33	155	26	15	33
	Nucleic acid binding transcription factor activity	0	0	1	1	0	1	0	0	0
	Antioxidant activity	1	3	2	1	2	2	0	0	0
	Molecular function regulator	1	2	4	2	0	7	1	0	1
	Protein binding transcription factor activity	0	0	0	0	0	0	0	0	0
	Structural molecule activity	7	9	18	4	3	10	0	1	2
	Transporter activity	8	14	19	7	4	18	4	3	3
**Biological process**	Developmental process	0	0	1	0	0	1	0	0	0
	Biological adhesion	0	3	5	3	1	3	0	0	0
	Metabolic process	68	106	169	94	40	169	42	17	37
	Single-organism process	30	59	104	50	25	98	16	9	21
	Immune system process	0	0	1	0	0	1	0	0	0
	Locomotion	0	0	0	0	0	0	0	0	0
	Localization	5	19	26	11	3	27	2	5	4
	Response to stimulus	0	3	10	3	0	7	1	0	0
	Signaling	0	3	8	3	0	6	1	0	0
	Cellular process	28	63	113	49	19	96	12	7	13
	Multi-organism process	0	0	0	0	0	0	0	0	0
	Biological regulation	3	15	23	9	6	18	2	1	0
	Cellular component organization or biogenesis	0	4	8	7	0	9	1	0	0

### Classification of the Identified Proteins Based on Subcellular Location

The amount of differentially expressed proteins in each subcellular location was determined according to the subcellular location annotation of the identified proteins (Table 5 and Supplementary Table S5). Low-temperature treatments induced the increase of proteins associated with the plasma membrane, cytosol, mitochondria, nuclear cytosol, as well as extracellular and nuclear proteins. Interestingly, new proteins were found in the peroxisome, plasma membrane, and mitochondria, as well as new extracellular and nuclear proteins in both GH2 and GH1 under cold stress conditions.

**Table 5:** The subcellular location of different proteins of nine groups.

Subcellular location	Differential expressed protein numbers								
	GH2-28 vs. GH1-28	GH2-16 vs. GH2-28	GH2-16 vs. GH1-16	GH2-R vs. GH2-28	GH2-R vs. GH2-16	GH2-R vs. GH1-R	GH1-16 vs. GH1-28	GH1-R vs. GH1-28	GH1-R vs. GH1-16
Cytosol, plasma membrane	0	0	0	0	0	0	0	0	0
Cytosol, mitochondria	1	1	2	1	0	2	0	0	0
Cytosol, nuclear	4	10	18	8	2	11	3	1	2
Mitochondria, nuclear	0	0	1	0	0	0	0	0	0
Cytosol, peroxisome	0	0	0	0	0	0	0	0	0
Nuclear	13	28	44	20	5	41	9	5	6
Plasma membrane	17	38	41	27	17	36	12	6	8
Lysosome	0	0	0	0	0	0	0	0	0
Endoplasmic reticulum, Golgi apparatus	0	0	0	0	0	0	0	0	0
Endoplasmic reticulum	6	6	7	5	2	6	2	3	3
Golgi apparatus	1	4	4	1	3	2	1	0	1
Peroxisome	0	2	1	3	0	1	0	0	0
Cytosol	27	63	110	55	27	105	15	10	20
Extracellular	35	82	105	74	26	89	32	20	23
Extracellular, plasma membrane	0	0	1	0	0	0	1	0	0
Cytoskeleton	2	2	1	1	2	3	0	1	0
Endoplasmic reticulum, mitochondria	0	0	0	0	0	0	0	0	0
Mitochondria	24	45	78	44	21	69	17	9	14

### Functional Enrichment of Differentially Quantified Proteins

**FIGURE 3:**
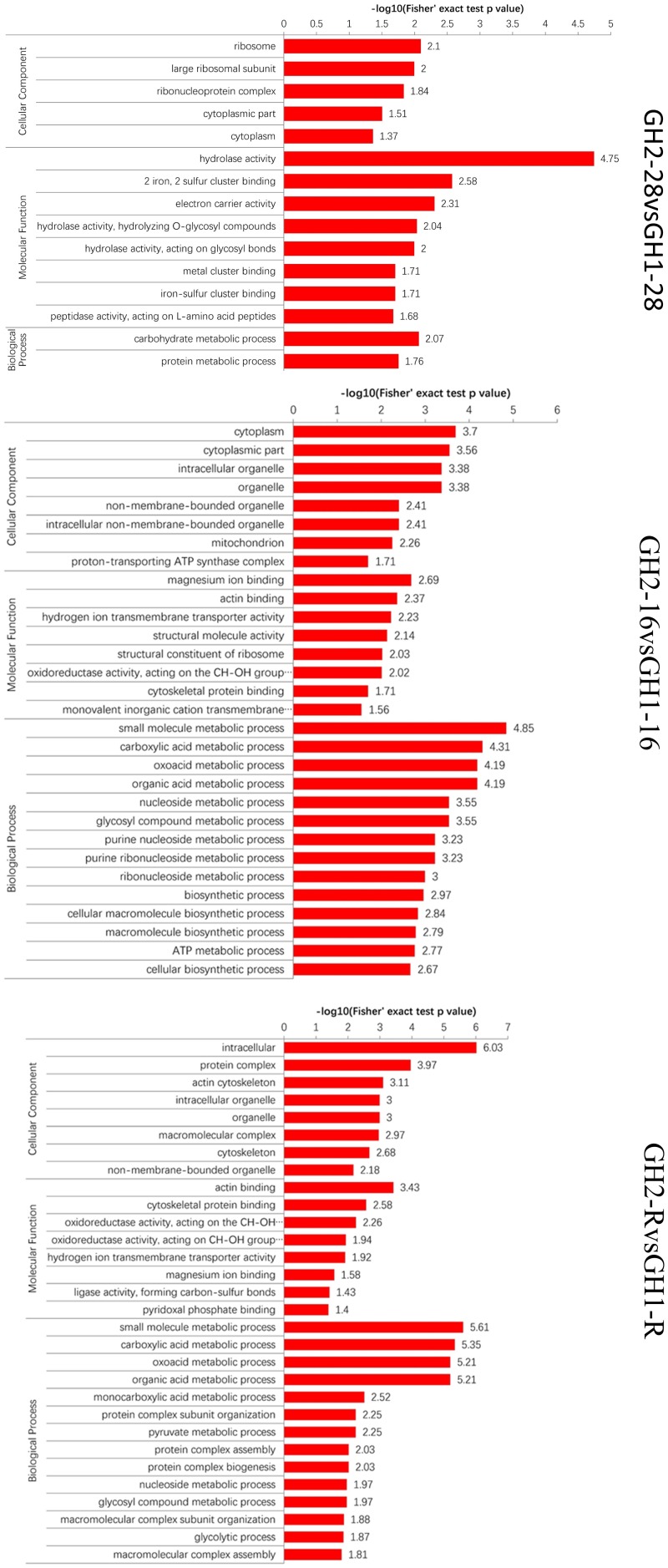
GO-based enrichment analysis of all the proteins.

After the proteins were assigned to different categories, the quantities were calculated via the −log10 (*p*-value) method (Figure 3 and Supplementary Table S6). Under normal temperature (GH2-28 vs. GH1-28), the difference in protein content varied from 1.37 for cytoplasm to 4.75 for hydrolase activity. After cold treatments (GH2-16 vs. GH1-16), the difference in protein content varied from 1.56 for monovalent inorganic cation transmembrane transporter activity to 4.85 for small-molecule metabolic processes. During the recovery phase (GH2-R vs. GH1-R), the difference in protein content varied from 1.4 for pyridoxal phosphate binding to 6.03 for intracellular proteins. These results indicate that during cold treatment and the recovery process, the number of proteins and protein contents increased both in GH1 and GH2.

### Functional Enrichment-Based Clustering for Comparable Groups

**FIGURE 4:**
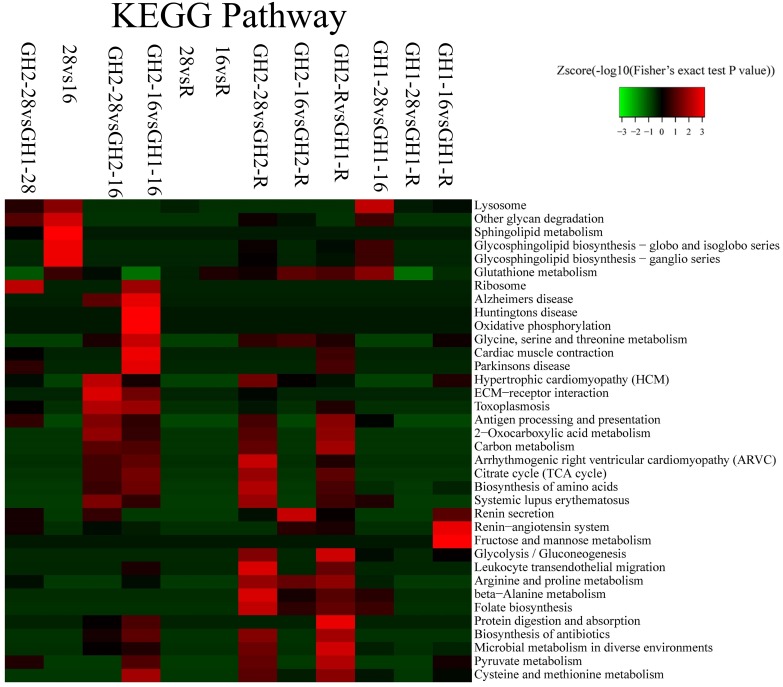
KEGG pathway enrichment-based clustering analysis of all the identified proteins.

After a GO-based enrichment analysis of all the proteins, KEGG pathway enrichment-based clustering analysis was employed to compare all the changes among GH1 and GH2 under the different treatments (Figure 4 and Supplementary Table S3). Significant changes were observed in oxidative phosphorylation, glycine, serine, and threonine metabolism, and in cardiac muscle contraction under cold stress (GH2-16 vs. GH1-16). In GH2, a low temperature caused changes in proteins associated with hypertrophic cardiomyopathy (HCM), extracellular matrix (ECM)-receptor interaction, toxoplasmosis, and antigen processing (GH2-28 vs. GH2-16). On the contrary, in GH1, a low temperature caused protein changes in the lysosome, with other glycan degradation proteins, in glycosphingolipid biosynthesis, and in glutathione metabolism (GH1-28 vs. GH1-16).

**FIGURE 5:**
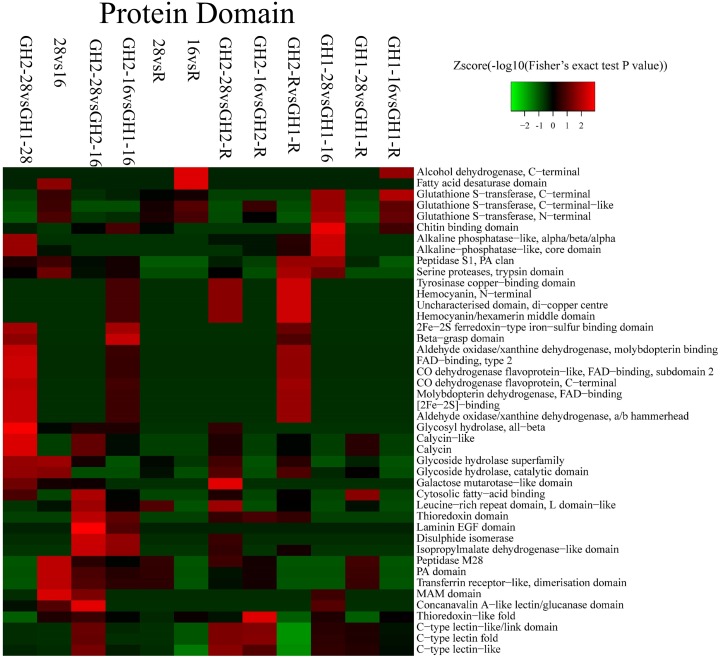
Protein domain of proteins identified in each treatment group.

Protein domains were analyzed to further explore specific protein families (Figure 5 and Supplementary Table S3). Significant changes were observed in proteins with 2Fe-2S ferredoxin-type iron-sulfur-binding domains, with beta-grasp domains, in aldehyde oxidase/xanthine dehydrogenases, and with molybdopterin binding under cold stress (GH2-16 vs. sGH1-16). In GH2, a low temperature caused changes in proteins with leucine-rich repeat (LRR) domains, L domain-like is one kind of protein with thioredoxin domains, disulphide isomerases, and laminin EGF domains (GH2-28 vs. GH2-16). On the contrary, in GH1, a low temperature caused changes in glutathione S-transferases (GSTs), glycoside hydrolase superfamily, PA domain, and C-type lectin (GH1-28 vs. GH1-16). These results corresponded with that of biological processes and cellular components.

### Validation of Proteomic Data by qRT-PCR Analysis

**FIGURE 6:**
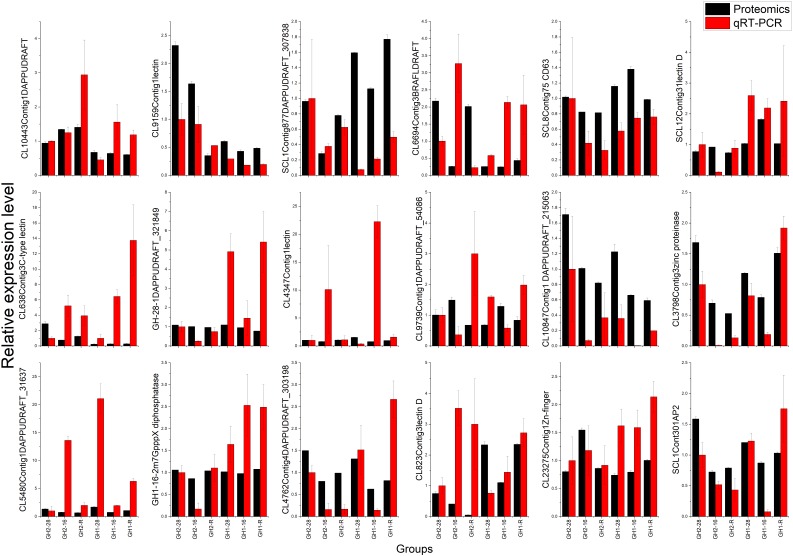
Validation of proteomic data by qRT-PCR analysis. The protein accession number and full names were: SCL8Contig75, tetraspanin-like protein CD63; CL3798Contig3, zinc proteinase; GH1_16_2-c14615_g2_i2, m7GpppX diphosphatase; SCL1Contig301, AP-2 complex subunit alpha; CL23275Contig1, Zn-finger in Ran-binding protein and other domain-containing proteins; CL9159Contig1, lectin 1; SCL12Contig31, lectin D1; CL638Contig3, C-type lectin; CL4347Contig1, lectin 2; CL823Contig3, lectin D2; CL10443Contig1, hypothetical protein DAPPUDRAFT_240263; SCL1Contig877, hypothetical protein DAPPUDRAFT_307838; CL6694Contig3, hypothetical protein BRAFLDRAFT_206907; GH1_28_1-c16133_g2_i1, hypothetical protein DAPPUDRAFT_321849; CL9739Contig1, hypothetical protein DAPPUDRAFT_54086; CL10847Contig1, hypothetical protein DAPPUDRAFT_215063; CL5480Contig1, hypothetical protein DAPPUDRAFT_31637, partial; CL4762Contig4, hypothetical protein DAPPUDRAFT_303198.

To validate the proteomic data, we performed the qRT-PCR analysis of 18 genes belonging to four groups: enzymes (i.e., tetraspanin CD63, m7GpppX pyrophosphatase, and zinc proteinase), transcription factors (i.e., TF, AP2, and zinc-finger), lectin family proteins, and DAPPUDRAFT family proteins. The mRNA expression of 18 genes corresponded to 18 proteins that were identified as differentially expressed in GH1 and GH2 under cold stress (Figure 6 and Supplementary Table S7). The proteomic and qRT-PCR data exhibited the same trends in most proteins such as lectin 1 (CL9159Contig1) and zinc-finger in Ran-binding protein and other domain-containing proteins (CL23275Contig1). These data indicate that the proteomic data were reliable and could be used for future studies. Furthermore, we summarized the domains of differentially expressed proteins in all the nine compared groups (Table 6). There were 18 proteins with significant differences between GH2-28 vs. GH1-28. However, under cold stress and under the recovery phase, differentially expressed proteins with significant differences included lectin, those with 2Fe-2S ferredoxin-type iron-sulfur-binding domains, alcohol dehydrogenase, GST, and LRR domains, among others.

**Table 6:** The subcellular location of different proteins of nine groups.

	Differential expressed protein domains								
Number	GH2-28 vs. GH1-28	GH2-16 vs. GH2-28	GH2-16 vs. GH1-16	GH2-R vs. GH2-28	GH2-R vs. GH2-16	GH2-R vs. GH1-R	GH1-16 vs. GH1-28	GH1-R vs. GH1-28	GH1-R vs. GH1-16
1	[2Fe-2S]-binding	Concanavalin A-like lectin/glucanase domain	2Fe-2S ferredoxin-type iron-sulfur binding domain	C-type lectin fold	C-type lectin fold	[2Fe-2S]-binding	Alkaline phosphatase-like, alpha/beta/alpha	C-type lectin fold	Alcohol dehydrogenase, C-terminal
2	2Fe-2S ferredoxin-type iron-sulfur binding domain	C-type lectin fold	Beta-grasp domain	C-type lectin-like	C-type lectin-like	2Fe-2S ferredoxin-type iron-sulfur binding domain	Alkaline-phosphatase-like, core domain	C-type lectin-like	C-type lectin fold
3	Aldehyde oxidase/xanthine dehydrogenase, a/b hammerhead	C-type lectin-like	CO dehydrogenase flavoprotein, C-terminal	C-type lectin-like/link domain	C-type lectin-like/link domain	Aldehyde oxidase/xanthine dehydrogenase, a/b hammerhead	Chitin binding domain	C-type lectin-like/link domain	C-type lectin-like
4	Aldehyde oxidase/xanthine dehydrogenase, molybdopterin binding	C-type lectin-like/link domain	C-type lectin-like	Galactose mutarotase-like domain	Thioredoxin-like fold	Aldehyde oxidase/xanthine dehydrogenase, molybdopterin binding	C-type lectin fold	Leucine-rich repeat domain, L domain-like	C-type lectin-like/link domain
5	Alkaline phosphatase-like, alpha/beta/alpha	Cytosolic fatty-acid binding	C-type lectin-like/link domain	Glycoside hydrolase superfamily		CO dehydrogenase flavoprotein, C-terminal	C-type lectin-like		Glutathione S-transferase, C-terminal
6	Alkaline-phosphatase-like, core domain	Disulphide isomerase	Leucine-rich repeat domain, L domain-like	Leucine-rich repeat domain, L domain-like		CO dehydrogenase flavoprotein-like, FAD-binding, subdomain 2	C-type lectin-like/link domain		Glutathione S-transferase, C-terminal-like
7	Beta-grasp domain	Isopropylmalate dehydrogenase-like domain				FAD-binding, type 2	Glutathione S-transferase, C-terminal		Glutathione S-transferase, N-terminal
8	Calycin	Laminin EGF domain				Hemocyanin, N-terminal	Glutathione S-transferase, C-terminal-like		
9	Calycin-like	Leucine-rich repeat domain, L domain-like				Hemocyanin/hexamerin middle domain	Glutathione S-transferase, N-terminal		
10	CO dehydrogenase flavoprotein, C-terminal	MAM domain				Leucine-rich repeat domain, L domain-like	Peptidase S1, PA clan		
11	CO dehydrogenase flavoprotein-like, FAD-binding, subdomain 2	Thioredoxin domain				Molybdopterin dehydrogenase, FAD-binding	Serine proteases, trypsin domain		
12	C-type lectin-like	Thioredoxin-like fold				Peptidase S1, PA clan			
13	C-type lectin-like/link domain	Transferrin receptor-like, dimerization domain				Serine proteases, trypsin domain			
14	FAD-binding, type 2					Tyrosinase copper-binding domain			
15	Glycoside hydrolase superfamily					Uncharacterized domain, di-copper center			
16	Glycoside hydrolase, catalytic domain								
17	Glycosyl hydrolase, all-beta								
18	Molybdopterin dehydrogenase, FAD-binding								

## Discussion

Pacific white shrimp are native to tropical areas, but they are now widely cultivated in subtropical areas. Pacific white shrimp can be raised for 2–3 cycles each year in southern China, whereas only one growth cycle can occur in northern China due to temperature limitations. Therefore, to extend the breeding cycle of these shrimp in northern China, it is necessary to breed cold-resistant cultivars to reduce death rates caused by cold stress. Suppression-subtractive hybridization (Peng et al., 2016) and transcriptomic studies (Long et al., 2013; Chopra et al., 2015; Wang et al., 2015) of shrimp and zebrafish have been carried out to better understand the molecular mechanisms involved in cold tolerance in aquatic organisms. Recently, proteomic techniques were extensively used in research associated with human disease (Zhu et al., 2018), protein o-glycosylation (You et al., 2018), and apicomplexan biology (Yakubu et al., 2018). Little is known about the proteome of Pacific white shrimp (Lu et al., 2016), especially under cold stress. Here, we carried out a comprehensive study of variations in proteins in a cold-tolerant cultivar (GH2) and a cold-sensitive cultivar (GH1) under low-temperature treatments. The hepatopancreas performs some of the same functions that the pancreas and the liver perform in humans; therefore, it is often used as an indicator of organismal health and for the nutritional, metabolic, and disease status in shrimp (Shekhar et al., 2013; Zhang et al., 2014). The hepatopancreas has also been used in detecting cold responsive genes and proteins (Fan et al., 2016; Peng et al., 2016). Here, the results demonstrated significant differences between the expressed proteins in the cold-tolerant and cold-sensitive cultivars, and these protein functions were largely associated with metabolic processes, cellular processes, single-organism processes, localization, biological regulation, and in response to stimuli. Heat shock proteins have previously been found in *Drosophila melanogaster* (Burton et al., 1988; Fujikake et al., 2005; Colinet et al., 2010), quail spleen (Ren et al., 2018), and chicken hearts (Zhao et al., 2013), under cold stress conditions. However, proteomics data reported herein did not indicate that heat shock proteins were involved in the cold response in the hepatopancreas of white shrimp. These may be due to the functions of the heat shock proteins in acute cold response (Leandro et al., 2004). Here, the cold-tolerant cultivar GH2 was reared for cold adaption. Based on proteomics and qRT-PCR results, we speculate that the following are the main mechanisms of cold tolerance in Pacific white shrimp.

### Functional Enzymes Participate in Cold Tolerance

In *Panagrolaimus davidi*, cold stress can induce the expression of trehalose-6-phosphate phosphatase, superoxide dismutase, and glutathione peroxidase (Thorne et al., 2014). Similar results were observed in *Camellia sinensis* during cold acclimation (Wang et al., 2013) and in Antarctic notothenioid fish (Chen et al., 2008). Typically, a cold signal will first lead to damages to the plasma membrane. Next, mitogen-activated protein kinases (MAPKs) and serine/threonine kinases are phosphorylated and enter the nucleus where various transcription factors, enzymes, and other proteins that modulate cellular activities are phosphorylated (Fujiwara and Denlinger, 2007; Wang et al., 2017). Our results indicate that free radical stress scavenging enzymes played key roles in white shrimp under cold stress in these experiments, consistent with studies in other species. For example, cold treatment has been shown to cause significant changes in GST (Tierbach et al., 2018), LRR domains, tetraspanin CD63 (Li G. et al., 2014; Chaiyadet et al., 2017), m7GpppX pyrophosphatase (Malys et al., 2004), zinc proteinase (Loewy et al., 1993), and different types of lectin protein families (Figures 5, 6). GSTs play important roles in insecticide/drug resistance and stress response in insects (Sookrung et al., 2018), rats (Wang et al., 2018; Yang et al., 2018), and the plant species *Arabidopsis thaliana* (Banday and Nandi, 2018). In addition, similar results were found in 2D-electrophoresis proteomics studies, i.e., GST Mu 3-like was upregulated more than 1.5-fold under cold stress in white shrimp (Fan et al., 2016). Overall, the data suggest that more stress response and detoxing proteins and enzymes were synthesized under cold treatments in the cold-tolerant cultivar GH2.

### Transcriptional Regulation Was Involved in Cold Stress Response

Transcription factors were highly accumulated in the cold-tolerant cultivar GH2. LRR proteins are known to be involved in cold stress response in plants (Meyer et al., 1999; Yang et al., 2014). In humans, LRR proteins are considered to be related to Parkinson’s disease (Kett and Dauer, 2012) and lipid rafts (Hatano et al., 2007). Our results suggested that LRR transcription factors may play roles in lipid membrane protection resulting in cold tolerance in GH2. We found that AP2 and zinc-finger transcription factors were highly expressed in GH2-28 and GH2-16 compared to those of GH1-28 and GH1-16. AP2 interaction with (G/a) (C/t)CGAC motif (Xue, 2002) has been confirmed to participate in cold tolerance in plants (Kang et al., 2011; Du et al., 2016). Zinc-finger transcription factors have been shown to be involved in the response to cold acclimation in catfish (Ju et al., 2002), bees (Xu et al., 2017), and rice (Bai et al., 2015). Cold-responsive genes, such as heat shock proteins (HSPs) and serine/threonine kinases (STKs) and especially rely on the lectin type, were significantly upregulated through transcriptional regulation (Xu et al., 2017).

### Lectin and DAPPUDRAFT (Alkaline Phosphatase) Protein Families Played Versatile Roles in Cold Tolerance

On the structural level, the lectin protein family is tightly related to cold tolerance (Moulessehoul et al., 1992; Gronwald et al., 1998). Interestingly, the function of lectin proteins relies on alpha1,3-galactosyltransferase (Khraltsova et al., 2000), cytoskeleton (Timofeeva et al., 2000), and especially rely on the lectin type (Meng et al., 2017; Hung et al., 2018). In this study, three C-type lectin proteins were differentially expressed under the different temperature treatments (Figure 5), suggesting that each individual lectin played a specific role in cold tolerance in this organism. Interestingly, the protein expression levels observed from the proteomic analysis were similar to those observed in the qRT-PCR analysis (Figure 6). DAPPUDRAFT has been identified as an alkaline phosphatase (EC3.1.3.1) protein family in the water flea (Colbourne et al., 2011). Alkaline phosphatases are key enzymes in sea bream fish that are involved in cold tolerance (Mateus et al., 2017). These enzymes have also shown to have protective roles in liver (Bruinsma et al., 2015; Hjorleifsson and Asgeirsson, 2016), bone (Mateus et al., 2017), and ischemia-reperfusion injuries in rats (Li R. et al., 2014). Our analyses found that the expression levels of several DAPPUDRAFT proteins (240262, 307838, and 206907) were higher in GH2 than in GH1, suggesting that the cold tolerance of GH2 was related to alkaline phosphatase activity, most likely due to the fact that these enzymes can help protect the liver, bones, and blood of shrimp from cold-stress damage. Interestingly, not all the cold-responsive gene expressions that procede protein synthesis, and this could be due to a lack of energy for modification at post-transcriptional and post-translational levels, such as glycosylation (Yakubu et al., 2018; You et al., 2018).

It is well known that qRT-PCR is an effective way to perform quantitative proteomic analysis (Yan et al., 2006; Fan et al., 2013; Zhang et al., 2016), and there are five types of expression patterns at the qRT-PCR and protein level, namely: (A) upregulated at both the transcriptional and protein level, (B) upregulated at the transcriptional level but downregulated at the protein level, (C) downregulated at the transcriptional level but upregulated at the protein level, (D) no change at the transcriptional level but upregulated at the protein level, and (E) no change at the transcriptional level but downregulated at the protein level (Zhang et al., 2016). Expressions of most of the lectin genes exhibited the same patterns as the protein expression (Table 6 and Figure 6), suggesting that the key proteins involved in cold-tolerance in GH2 were lectin and phosphatase, among others. Based on the findings from previous studies, it can be speculated that cold stress first leads to the damage of the plasma membrane (Takahashi et al., 2014) that will induce signal cascades by phosphatase (Kristensen et al., 2016), zinc proteinase, and m7GpppX diphosphatase. Through a second messenger (Zieger et al., 2011; Kocharunchitt et al., 2012) or hormone synthesis (Cerny et al., 2014) at the transcriptional level, the AP-2 transcription complex and zinc-finger transcription factors (Koehler et al., 2012) regulate the expression of functional genes, including different lectin and DAPPUDRAFT proteins (Figures 5, 6). After translation and regulation at the post-translational level (Chen et al., 2014), proteins are synthesized and used for the cold stress response in Pacific white shrimp.

## Ethics Statement

This study was carried out in accordance with the recommendations of the guidelines of the Ministry of Agriculture and Rural Affairs of the China.

## Author Contributions

J-XP and X-LC analyzed and interpreted the data and drafted the manuscript. J-XP, P-PH, P-YW, BZ, Y-ZZ, and Q-YL set the experimental design and coordinated the study. J-XP, P-PH, and P-YW performed the proteome study. XC, MP, D-GZ, and C-LY assisted with proteomic analysis.

## Conflict of Interest Statement

The authors declare that the research was conducted in the absence of any commercial or financial relationships that could be construed as a potential conflict of interest.

## Footnotes

1 http://www.ebi.ac.uk/GOA/

2 http://www.ebi.ac.uk/interpro/search/sequence-search

3 https://wolfpsort.hgc.jp/

4 https://www.genome.jp/kegg/

5 https://www.ebi.ac.uk/interpro/

6 http://www.iprox.org/

## Supplementary Material

TABLE S1 Protein amino sequences in proteomics analysis of GH1 and GH2 under different temperature treatments.

TABLE S2 Protein accession and description of 3,349 identified proteins in proteomics analysis of GH1 and GH2 under different temperature treatments.

TABLE S3 Differentially expressed up-regulated and down-regulated proteins of GH1 and GH2 under different temperature treatments.

TABLE S4 GO annotation information of identified proteins of GH1 and GH2 under different temperature treatments.

TABLE S5 Subcellular location of differentially expressed proteins of GH1 and GH2 under different temperature treatments.

TABLE S6 Proteins GO Catalogs and quantities of GH1 and GH2 under different temperature treatments.

TABLE S7 18 genes qRT-PCR data and corresponding 18 proteins quantities.
